# Enzyme-Responsive Hydrogels as Potential Drug Delivery Systems—State of Knowledge and Future Prospects

**DOI:** 10.3390/ijms23084421

**Published:** 2022-04-16

**Authors:** Marcin Sobczak

**Affiliations:** 1 Department of Biomaterials Chemistry, Chair of Analytical Chemistry and Biomaterials, Faculty of Pharmacy, Medical University of Warsaw, 1 Banacha St., 02-097 Warsaw, Poland; marcin.sobczak@wp.pl or marcin.sobczak@wum.edu.pl; Tel.: +48-22-572-07-83; 2 Military Institute of Hygiene and Epidemiology, 4 Kozielska St., 01-163 Warsaw, Poland

**Keywords:** biomedical hydrogels, stimuli-responsive hydrogels, enzyme-responsive hydrogels, drug delivery systems, controlled release

## Abstract

Fast advances in polymer science have provided new hydrogels for applications in drug delivery. Among modern drug formulations, polymeric type stimuli-responsive hydrogels (SRHs), also called smart hydrogels, deserve special attention as they revealed to be a promising tool useful for a variety of pharmaceutical and biomedical applications. In fact, the basic feature of these systems is the ability to change their mechanical properties, swelling ability, hydrophilicity, or bioactive molecules permeability, which are influenced by various stimuli, particularly enzymes. Indeed, among a great number of SHRs, enzyme-responsive hydrogels (ERHs) gain much interest as they possess several potential biomedical applications (e.g., in controlled release, drug delivery, etc.). Such a new type of SHRs directly respond to many different enzymes even under mild conditions. Therefore, they show either reversible or irreversible enzyme-induced changes both in chemical and physical properties. This article reviews the state-of-the art in ERHs designed for controlled drug delivery systems (DDSs). Principal enzymes used for biomedical hydrogel preparation were presented and different ERHs were further characterized focusing mainly on glucose oxidase-, β-galactosidase- and metalloproteinases-based catalyzed reactions. Additionally, strategies employed to produce ERHs were described. The current state of knowledge and the discussion were made on successful applications and prospects for further development of effective methods used to obtain ERH as DDSs.

## 1. Introduction

One of the strategies to improve the biosafety and effectiveness of therapy is the use of a so-called intelligent carriers of medicinal substances. Therefore, one of the priority goals of modern pharmacy and biomedicine is the development of new solutions in the field of drug delivery systems (DDSs) [1,2]. 

**Figure 1:**
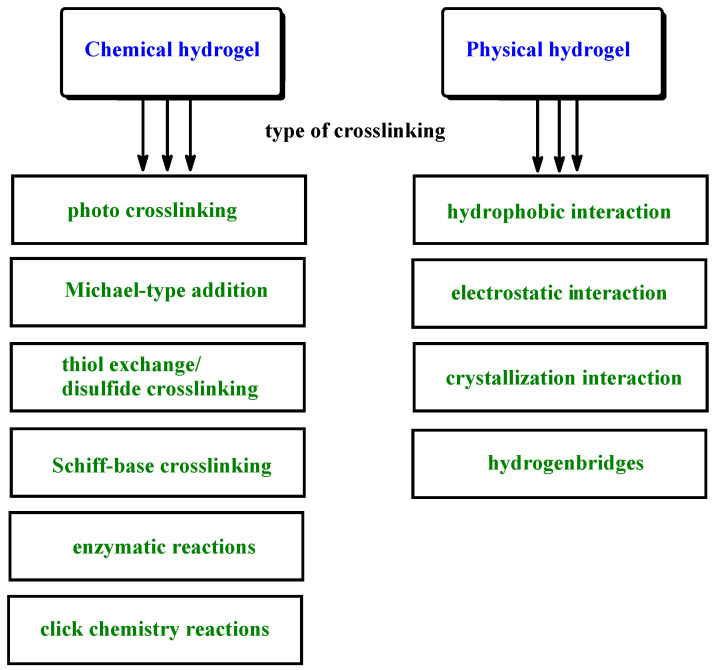
Chemical and physical hydrogels – type of cross-linking techniques.

Among a great variety of known types of DDSs hydrogels constitute a very interesting and promising group. Indeed, hydrogels-based DDSs open many opportunities for effective therapeutic delivery and monitoring. Most hydrogels possess biological traits, such as high tissue-like water content and permeability either for nutrients’ influx or metabolites’ excretion. Furthermore, they are characterized by good sorption abilities, biocompatibility, relatively high physical and chemical resistance under physiological conditions, similarity to human tissues, often sensitivity to environmental conditions as well as biodegradability. They have a special ability to swell and shrink in the aquatic environment without any significant, irreversible damage to their internal structure. In addition, a low interfacial tension, which prevents the absorption of proteins from body fluids, is also observed [3,4,5]. Hydrogel DDSs can be produced from natural, semi-synthetic and synthetic polymers [6,7]. However, there are also hybrid systems which combine the features of the above-mentioned groups of materials. Natural polymers used in the technology of hydrogel DDSs include e.g., alginate (ALG), cellulose, chitosan, chondroitin sulfide, collagen, cyclodextrin, gelatin (Gel), heparin, pectins and pullulan. In turn, the synthetic ones include mainly aliphatic polyesters, polyethers, copolyesters, polyurethanes or poly(organic phosphazenes). Due to the type of interactions between polymer chains, they can be divided into chemical and physical hydrogels. Chemical hydrogels are formed by covalent networks and do not dissolve in water without breakage of covalent bonds. The chemical cross-linking takes place by the photo cross-linking, Michael-type addition, thiol exchange/disulfide cross-linking, Schiff-base cross-linking, enzymatic reactions or click chemistry reactions (e.g., cross-linked gelatin, albumin, polysaccharides, poly(vinyl alcohol)). On the other hand, physical hydrogels are formed by dynamic cross-linking of synthetic or natural building blocks based on non-covalent interactions such as hydrophobic, electrostatic or crystallization interaction and hydrogenbridges (e.g., poly(acrylic acid), poly(methacrylic acid), poly(ethylene glycol), dextran, chitosan, carboxymethyl curdlan, pullulan, poly(vinyl alcohol) (Figure 1). 

**Figure 2:**
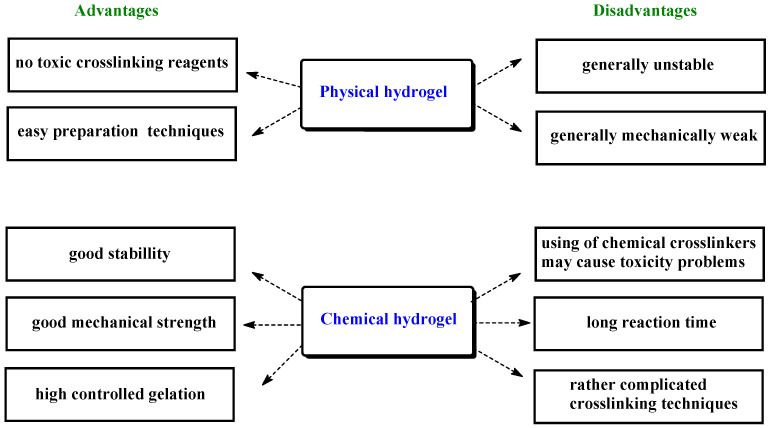
Advantages and disadvantages of chemical and physical hydrogels.

Importanlly, the preparation of physically cross-linked hydrogels does not require a cross-linking agent, which is important in the context of the toxicity of the system. Chemical hydrogels are often more physiologically resistant and have better mechanical properties than physical hydrogels (Figure 2). [3,8,9,10,11,12,13].

From the point of view of biomedical applications, the most interesting seems to be polymer gels, which undergo a reversible change in their volume under the influence of external factors. They are called intelligent biomaterials or smart hydrogels [3,4,14,15]. The phase transition of smart hydrogels can be triggered by a number of stimuli. The stimulus may be physical (temperature, magnetic field and light), chemical (pH, ionic strength) or the presence of specific chemical compounds (natural or synthetic) and enzymes. The phase transition may take place continuously within a certain range of the environmental parameters change or discontinuously through a step change in a volume. Hydrogels made of biodegradable polymers sensitive to the presence of specific enzymes have also a great potential in the DDSs technology (stimuli-responsive hydrogels-SRHs). The endogenous enzymes-induced breakdown of the polymer network gives the basis for the production of biodegradable carriers, where the drug substance is released in a specific place as a result of an enzymatic reaction [4,16].

Hydrogels sensitive to bioactive substances (HSBFs) are becoming more and more popular due to their potential application in the technology of various biomaterials and DDSs. The innovative generation of DDSs recognizing specific chemical compounds in the body and releasing the drug in response to their presence gives completely new possibilities to control the release of drugs, also at the target site of their action (in diseased tissue, population of pathological cells) [4,10,13,17]. HSBFs are systems which change their properties (i.e., erosion biodegradation, swelling/shrinking behavior) due to the presence, activity and concentration of specific biological factors. HSBFs are generally divided into following main groups: glucose-responsive, glutathione-responsive, specific enzyme or the presence of antibodies sensitive [4,18,19]. 

Enzyme-responsive materials (ERH) constitute a small, but extremely interesting group of HSBFs. The practical use of ERHs is an intensively increasing phenomenon in light of the inspiration for pharmaceutical applications [3,4]. 

This paper aims at presenting the state-of-the art in ERHs for controlled drug delivery applications. The basic knowledge of strategies for ERHs technology and potential application in therapy e.g., cancers or diabetes mellitus, have been described in details. I hope that this review will be widely useful for researchers, clinicists and technologists interested in new intelligent DDSs.

## 2. Enzyme Used in Biomedical Hydrogels Synthesis

**Figure 3:**
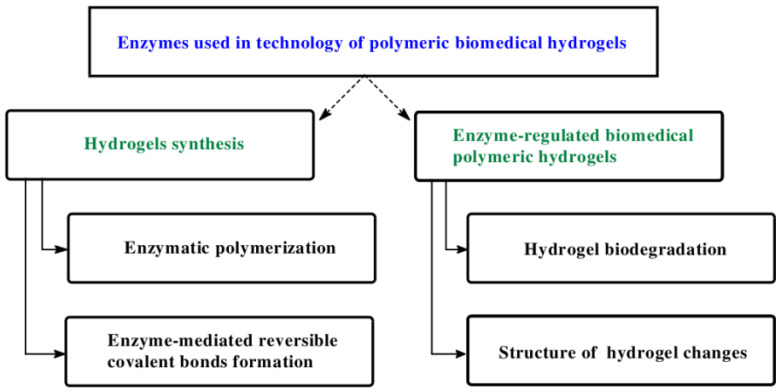
Enzymes used in technology of polymeric biomedical hydrogels.

Development of hydrogels formation methods using specific enzymes is a very interesting trend of scientific research. (Figure 3). The main approach for enzymatic fabrication of healable polymeric hydrogels is a self-assembly and polymerization method. By using enzymes, it is also possible to reversible covalent bonds (e.g., acylhydrazone, disulfide, borate ester, imide bonds), that can be cleaved under stimuli and subsequently re-formed, to endow hydrogels required properties. Moreover, the enzymatic degradation is important for the implanted or injected hydrogels to be autonomously cleared in a noninvasive manner. 

The following enzymes are constantly used in the preparation of hydrogels: elastase, horseradish peroxidase (HPR), trans-glutaminase (TGlu) and tyrosinase (Tyr) (Table 1). This is mainly driven by the need to develop gentle cross-linking strategies able to induce hydrogelation (i.e., cross-linking of the polymer) in vivo without damaging surrounding tissues. Such materials can find application as injectable scaffolds and DDSs, in particularly [20,21,22].

**Figure 4:**
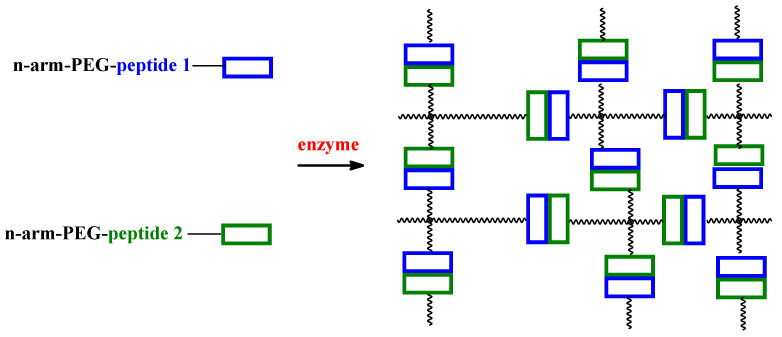
Enzyme-catalyzed hydrogel formation by cross-link two multiarm PEG-peptide conjugates.

ERHs usually contain side chains of amino acids that can be covalently connected by the enzyme. A great example is the TGlu sensitive system. This naturally (microbial) occurring cross-linking enzyme has been widely used to cross-link Gel, for example [30]. In addition, TGlu has also been used to cross-link a polypeptide with poly(ethylene glycol) (PEG) (Figure 4) [31,32]. Sanborn et. al designed a sophisticated system where the enzymatic cross-linking of a polymer-peptide conjugate was triggered thermally [27].

**Figure 5:**
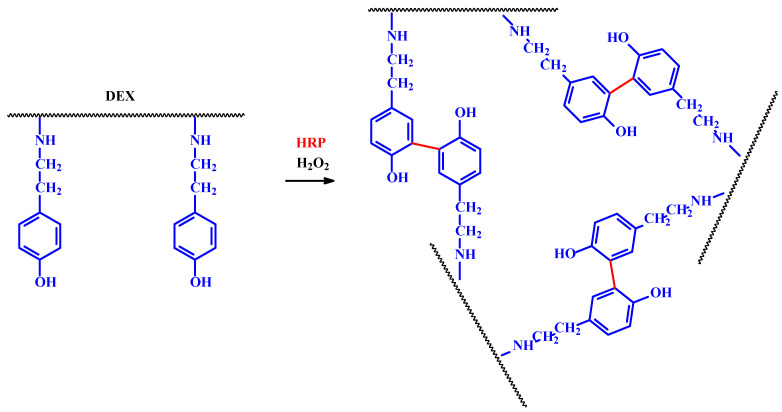
Enzymatic cross-linking of DEX–tyramine conjugates.

Another method to generate cross-links between polymer chains is the enzymatic conversion of side groups into more reactive species. They are subsequently able to react with moieties being located in neighboring polymer chains [20]. The method involving oxidative coupling of phenols using HRP in the presence of hydrogen peroxide, leading to the uprising a hydrogel built from hyaluronic acid and tyramine conjugates, has been made. It was observed that both the mechanical properties and degradation kinetics of the hydrogel depended on the amount of hydrogen peroxide used [35]. Another interesting example is hydrogels obtained from DEX and ALG modified with tyramine and subsequently cross-linked with HRP (Figure 5) [24,25,26]. Chen et al. used Tyr to convert tyrosine residues present in Gel to quinones. The obtained products were able to react with other amino acids from neighboring polypeptide chains. This method was also used to cross-link a mixed hydrogel consist of both Gel and chitosan [24,29,33,34,36]. 

**Figure 6:**
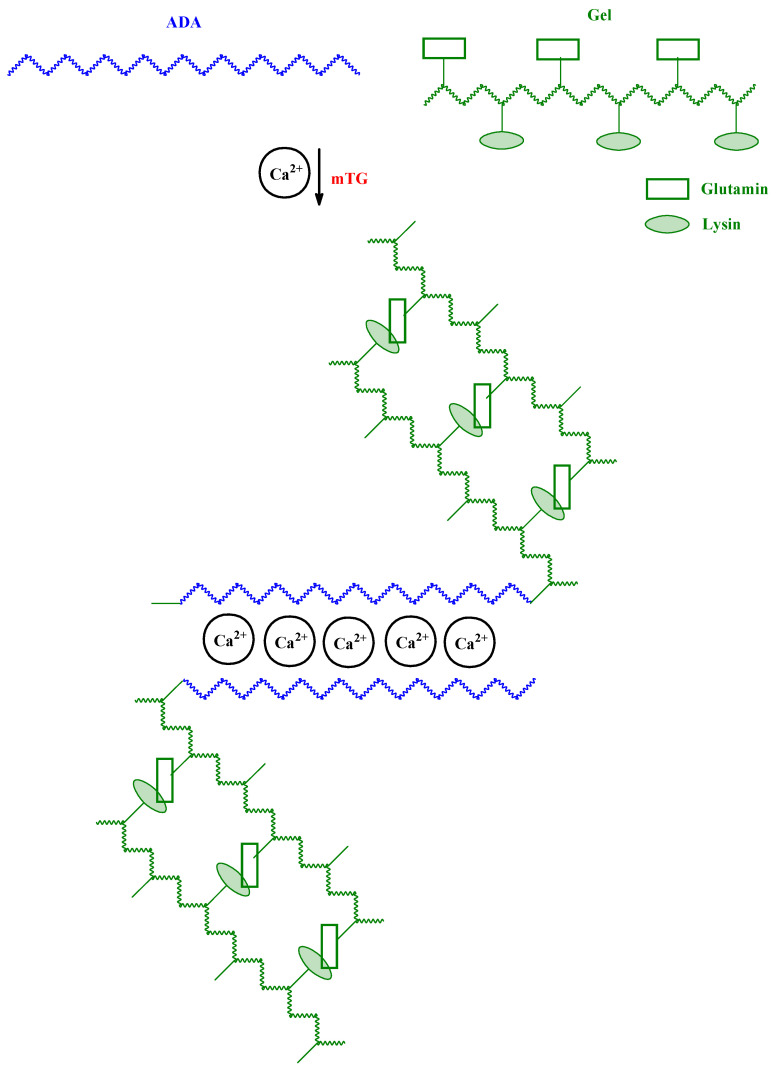
Microbial (mTG) and Ca^2+^ cross-linked ADA-Gel hydrogel.

A marine derived oxidized ALG, alginate dialdehyde (ADA), and Gel hydrogel systems (ADA-Gel) have been obtained [29]. The systems have been cross-linked via Ca^2+^ and microbial transglutaminase (mTG) interaction (Figure 6). It was possible to control the degradation behavior of the hydrogels to be stable for up to 30 days of incubation. The cytocompatibility of mTG cross-linked ADA-Gel was assessed using NIH-3T3 fibroblasts and ATDC-5 mouse teratocarcinoma cells. Both cell types showed highly increased cellular attachment on mTG cross-linked ADA-Gel in comparison to Ca^2+^ cross-linked hydrogels [29].

Enzymatic cross-linking emerged as an alternative tool to increase mechanical strength, which can be adjusted by the degree of enzymatic cross-linking. Tyramine-modified gellan gum (Ty-GG) hydrogels were developed via HRP crosslinking. It was found that obtained SRHs were characterized with either high mechanical strength or resistance. Ty-GG hydrogels also exhibited no cytotoxic effects and did not negatively affect the metabolic activity as well as proliferation of chondrogenic primary cells [29]. 

A new enzyme-mediated cross-linked hydrogel composed of silk sericin is proposed for the first time [37]. The developed hydrogel cross-linking strategy was performed via HPR, under physiological conditions. The hydrogels presented a high degree of transparency, mainly due to their amorphous conformation. Degradation studies revealed hydrogels stable in phosphate buffer solution (PBS) (pH 7.4) for 17 days, while in the presence of protease XIV (3.5 U/mg) and under acute and chronic physiological pH values, the stability decreased to 7 and 4 days, respectively. During protease degradation, the present sericin hydrogels demonstrated antioxidant activity. *In vitro* studies performed with L929 fibroblast cell line demonstrated that these hydrogels were found noncytotoxic, promoting cell adhesion and massive cell colonization after 7 days of culture, demonstrating that cells maintained their viability and proliferation. In addition, the application of sericin-based hydrogel, using in vivo diabetic wound model, validated the feasibility of the *in situ* methodology and demonstrated a local anti-inflammatory effect, promoting the healing process [37]. 

Enzymatic cross-linking of polymer-catechol (CAT) conjugates is a very interesting method to fabricate *in situ*-forming, injectable hydrogels, in the presence of HRP and H_2_O_2_ [28]. However, because of the occurrence of numerous variables such as polymer concentration, oxidizing agent/enzyme, and stoichiometry, the design of the polymer with optimized tissue adhesive property is still challenging. A poly(γ-glutamic acid)-dopamine (PGADA) conjugate was synthesized, and *in situ* hydrogels were fabricated via enzymatic cross-linking of a CAT moiety. Adhesive property, the gelation behavior and mechanical strength of the PGADA hydrogel were the effects exerted by various factors, such as polymer concentration, catechol substitution degree, HRP concentration, and H_2_O_2_ content [28].

## 3. Hydrogel Biodegradation

Polymer hydrogels can be biodegradable under the influence of a variety of enzymes (Table 2). As commonly known, enzymatic biodegradation of natural hydrogels is one of the most common processes during tissue remodeling. Due to this fact biodegradable SRHs frequently find application in the controlled release of drugs [22].

The first SRHs have been obtained from natural biodegradable polymers such as dextran and Gel [39,48]. These materials could be broken down by dextranase (Dextr) and α-chymotrypsin (α-Chym), respectively. Enzyme cleavable units could be also built into the polymer hydrogel [38,39,40].

Biodegradable interpenetrating polymer network (IPN) structured hydrogels consisting of Gel and DEX were prepared by sequential crosslinking reactions of Gel and methacryloylated-DEX [38]. It was found that phase separation of these hydrogels is above or below the sol-gel transition temperature (T_trans_) of Gel. Enzymatic degradation by either α-Chym or Dextr was hindered for the hydrogel prepared below T_trans_, whereas this hydrogel was perfectly degradable in the presence of both enzymes. Such a specific feature of enzymatic degradation was not observed for the hydrogel prepared above T_trans_. These results suggest that double-stimuli-responsive degradation of IPN-structured hydrogels is related to their phase separation [38].

Interesting strategy to produce enzyme degradable polymer hydrogels is using enzymatically cleavable cross-linkers. PEG-functionalized at both sides with short peptides that were terminated with polymerizable groups has been obtained, for example [44]. These hydrogels could be degraded with either matrix metalloprotease 1 (MMP-1) or plasmin (Plas). Another example of enzymatically degradable PEG-hydrogels is the elastase- and collagenase-SRHs prepared [42]. Moreover, the multi-armed PEG hydrogels cross-linked with short peptide strands that respond to MMP-1, Plas, trypsin and papain have been obtained [45,47,49,52,53,54,55,56,57]. Other peptide cross-linked MMP sensitive systems were prepared from Pluronic and copolymers of N-isopropylacrylamide and acrylic acid [22,46,50]. Poly(2-hydroxyethyl methacrylate)–PEG hydrogels have been obtained by Khelfallah et al. These hydrogels could be degraded by subtilisin via the cleavage of a peptide sequence incorporated into the polymer chain. 

Another strategy focuses on hydrogel preparation which characterized thermally control the their enzymatic degradation [41]. Polymerisable peptides, formed the basis of an α-Chym degradable hydrogels, have been synthesis. It was found that these biomaterials can be used to thermal control of polymer hydrogel enzymatic degradation. With reference to the above-mentioned, a composite material consisting of poly(N-isopro-pylacrylamide) (pNIPAM) grafted on DEX and a pNIPAM–N,N-dimethylacrylamide copolymer, that could be degraded by Dextr, was prepared. The biodegradation process was only between the cloud points of the two copolymer systems, effectively inhibiting enzymatic degradation below 30 °C and above 40 °C due to the steric hindrance of the enzyme [20,39,48].

Another concept of obtaining enzymatically degradable PEG-hydrogels is to change system hydrophilicity. For this purpose, modifications of side chains of poly(allylamine) with acetyl protected dialanine residues were suggested. Thereby, the polymer was less hydrophilic and caused the formation of a self-supporting polymer hydrogel [23]. The acyl group was removed by elastase restoring the polymers hydrophilicity and destroying the self-supporting hydrogel. 

The polymer hydrogels consisting exclusively of amino acids have also been obtained. One of the natural amino acid, aspartic acid, was polymerized to polysuccinimide which was cross-linked by a tetrapeptide sequence designed for proteolytic degradation, and then the corresponding poly(aspartic acid) hydrogel was obtained by alkaline hydrolysis. The hydrogel dissolved in the presence of trypsin (TRYP). According to in vitro cellular assays, the degradation products of the hydrogel cross-linked with the peptide were non-cytotoxic and non-cytostatic [51].

## 4. Enzyme-Responsive Hydrogels as Drug Delivery Systems

As already mentioned, ERHs such as DDSs seem to be fascinating because they are selective and capable to be activated by specific factors [4,58,59]. 

**Figure 7:**
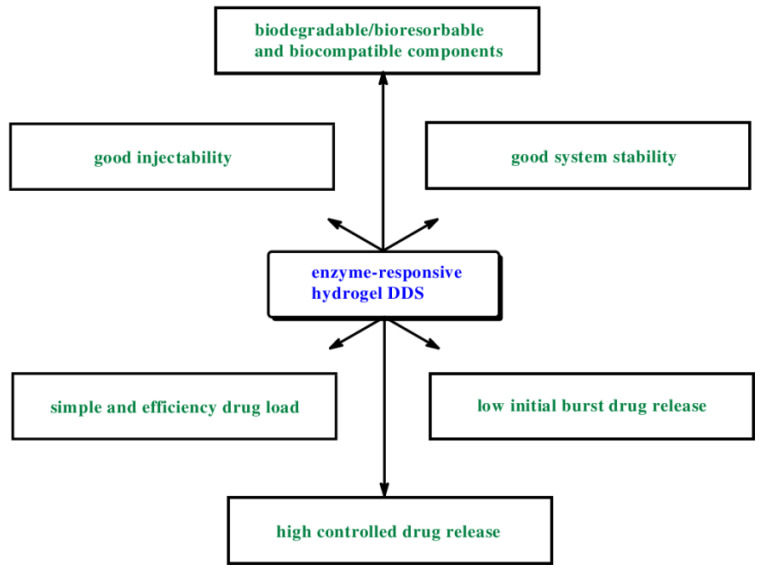
An ideal enzyme-responsive hydrogel drug delivery system.

Nonetheless, ERHs as DDSs must meet the following criteria (Figure 7):

- the hydrogel network must contain a chemical moiety being a substrate for the enzyme,
- chemical moieties need to be accessible to enzymatic active center,
- enzymatic reaction must cause significant change of hydrogel properties,
- the enzymatic cleavage of cross-linkers of hydrogel must lead to its biodegradation (or changing structures), and consequently drug release [4,58].

New ERHs as DDSs are mainly dedicated to the treatment of civilization diseases, particularly cancers and diabetes.

### 4.1. Enzyme-Responsive Hydrogels as Insulin Delivery Systems

As it is commonly well known, a great challenge in the field of HSBF is the research of various formulations that sense glucose levels and respond to deliver the right dose of insulin (Ins). This approach, referred to as fully synthetic pancreas, provides for closed-loop Ins therapy (that releases insulin in response to an appropriate level of glucose (Gluc) in the blood). Strategies for incorporating Gluc detection into preparations can be broadly classified into three subsets: enzymatic detection, natural Gluc-binding proteins, and synthetic molecular recognition, respectively [60,61].

Gluc sensitive hydrogels are very useful in the design and preparation of self-regulating Ins delivery systems (Table 3). In order to obtain this type of drug carriers, an approach consisting in the modification of pH-sensitive hydrogels with the enzyme glucose oxidase (GO) was used. Two mechanisms have been exploited in GO-incorporated hydrogels for Ins delivery: Gluc- triggered swelling/de-swelling or Gluc-triggered dissociation. This enzyme is capable of converting Gluc into gluconic acid (Gluc acid). Gluc caused a decrease in pH in the vicinity of the reaction environment, and as a consequence, Ins was released from the pH-sensitive hydrogel [17,18,19,62,63,64,65,66,67,68]. 

For example, hydrogels synthesized on the basis of N,N-diethylaminoethyl methacrylate and 2-hydroxypropyl methacrylate cross-linked with a polyacrylamide membrane (in which GO has been immobilized) have been obtained. In this type of DDS Gluc has diffused to the membrane. Conversion to Gluc acid took place with the participation of GO and the pH was lowered. The low pH of the membrane caused ionization (protonation) of the amino groups present in the DDS. This further led to the swelling of the hydrogel and the membrane permeability to Ins increased [62,63].

Another interesting example of GO-incorporated hydrogel for Ins delivery is poly(diethylaminoethyl-g-ethylene glycol) hydrogels which had a steep volume transition at pH 7.0. After incorporated with GO, the HSBF showed a pulsatile reversible volume change when glucose concentration varied between 0 and 2 g/L [66].

### 4.2. Enzyme-Responsive Hydrogels as Anticancer-Drug Delivery Systems

Continuous increase in cancer incidence is one of the greatest challenges of modern medicine. Despite the relatively large number of available treatments, the effectiveness of anti-cancer therapies is still unsatisfactory. The main difficulties in effective cancer therapy result from the emerging resistance of cancer cells to chemotherapy, restriction and/or inhibition of the intracellular transport of drugs, inactivation of active substances and high systemic and organ toxicity. As commonly known, tumor cells do not stay in homeostasis and their enzyme levels are dysregulated and differ from normal cells [58,77]. Currently used standard methods of anticancer drug administration incompletely use their therapeutic potential. The main problem is the biodistribution of the drug throughout the body, which reduces the chances of the appropriate dose of the drug reaching the target site. At the same time, a necessity to use much higher initial doses of the drug taken is observed. This in turn, can cause in the occurrence of many clinically important negative side effects to the therapy. 

One of the strategies of modern pharmacy is to obtain such dosage forms so that the drug directly reaches the cancer tissue/cells. Then the initial dose/intake of the drug could be limited and the toxic effects of its action on healthy tissues would be minimized. The various ways to achieve this goal include the use of hydrogel anti-cancer-DDSs (Table 3). They increase the probability of delivering the anticancer drug to the target site, eliminate its distribution throughout the body and enable its release with the assumed kinetics. It is then possible to maintain a constant therapeutic concentration of the drug while reducing its total dose. Hydrogel DDSs can also protect the delivered substances against various proteolytic enzymes. Importantly, they may easily increase drug stability in the physiological environment.

A very important and intensively developed area of research on ERH is materials which can be used in cancer therapy. Here, metalloproteinases (MMPs) can be of greatest importance. MMPs are a group of endopeptidases able to cleave peptide bonds. This group consists of 24 enzymes that play crucial roles in the metabolism. They are divided into gelatinases, collagenases, stromelysins and membrane-type MMPs. MMPs and their specific inhibitors serve as a significant elements involved in the cellular homeostasis. They are involved in adhesion, survival, proliferation and differentiation, migration and intercellular interactions etc. MMPs are promising biological triggers for enzyme-responsive antitumor DDSs. Cancer tissues show some disturbances of homeostasis, while MMPs enzymatic activity is significantly elevated. Moreover, tumor cells secrete MPPs and other proteolytical enzymes to extracellular matrix, decomposing it to make more space for tumor growth. MMPs-sensitive hydrogels are generally formed by cross-linking the polymeric chains with a specific, peptide-bound amino acid fragments, susceptible for MMPs activity. The enzymatic cleavage of these cross-linkers results in polymer biodegradation and further drug release [4,78,79]. 

In regard to the above-mentioned, MMPs-sensitive ERH containing PEG-coated magnetic iron oxide nanoparticles, able to selective doxorubicin (DOX) drug release, have been obtained [69]. MMPs sensitivity was provided by incorporation the specific peptide fragment (GGGPQG↓IWGQGK) (PQ), vulnerable to enzymatic cleavage [70]. The system was obtained by photoinitiator-coated nanoparticles reaction with acrylate-PEG-PQ–PEG-acrylate conjugates, PEG-diacrylate and acrylate-PEG-RGDS (RGDS fragment is a specific ligand for α_v_β_3_ integrin enables nanocarriers tumor targeting and endocytosis process). As a result of photopolymerization, the obtained nanomatrices have collagenase-degradable, cells targeting hydrogel shell. The conducted assay exhibited increased degradation of nanocarriers in collagenase-containing environment and DOX loading efficacy more than 97%. *In vitro* drug release from obtained hydrogel was 60% and 36% after 4 days, in a collagenase and non-collagenase release medium, respectively. Moreover, cytotoxicity of DOX-loaded targeted and non-targeted nanocarriers was evaluated by incubating with HeLa cells. It was found that targeted nanocarriers have sufficient cytotoxicity. The tests with healthy fibroblasts showed decreased cytotoxicity because of lower collagenase levels and limited integrin expression. As a control, the cells were treated by DOX-free nanocarriers, and the significant toxicity of hydrogel matrices was not observed. The prepared system seems to be interesting as theranostic, able to ensure controlled drug release and imaging due to efficient tumor cells uptake [70].

Dextranase (Dextr) has also been used to obtain biodegradable hydrogels that are sensitive to two types of enzymes simultaneously [38]. These hydrogels were composed of self-penetrating networks of PEG and DEX chains terminated with an oligopeptide. The second enzyme necessary for the biodegradation of the hydrogel network was papain. Interestingly, enzymatic degradation took place only when two enzymes were present at the same time. This type of double stimulation with enzymes allowed the precise anti-cancer drug release to neoplastic tissues, where the concentration of both enzymes was high [39].

Another approach involving the use of enzymes to deliver drugs within the large intestine is the use of azoreductases (Azored) produced by its microflora. Azoaromatic linkages that can be degraded by Azored have been used to obtain colon specific DDSs. These bonds were used in the cross-linking of pH-sensitive hydrogels. Hydrogels were synthesized from N,N-dimethylacrylamide, tert-butylacrylamide and acrylic acid, which, due to the presence of carboxyl groups, made the hydrogel sensitive to pH. It is worth emphasizing that due to the low degree of swelling in an acidic environment, this type of hydrogels protects protein drugs against digestion by protolytic enzymes in the stomach. As it is known, in further parts of the digestive system the pH is higher and the gels swell due to the ionization of the carboxyl groups contained in the hydrogel network. After reaching the colon, due to the presence of Azored, it is possible to degrade the hydrogel network and release the drug. Research on this type of carriers shows that the combination of enzymatic biodegradation and sensitivity to pH of hydrogels enables the delivery of drugs to target sites and their release via biodegradation mechanisms [80,81].

Among proteases mentioned above, there are enzymes able to cleave glycosidic bonds in carbohydrate structures. Hydrogels sensitive to the glycosidase activity in the context of anticancer therapy are used primarily as delivery systems of antineoplastic agents to the colon. The most widely studied are systems sensitive to β-mannanase, an enzyme presents in the small intestine. The backbone of the hydrogel is based on glucomannan or guar gum. The release takes place by cleavage of glycosidic bonds and hydrogel biodegradation [58].

ERHs as carriers of 5-fluorouracyl (5-FU) for colon-specific have been obtained [75]. Colon-specific DDs are efficient for increasing the availability of drugs at colon region and are desirable for the treatment of local diseases such as ulcerative colitis and colonic cancer. Hydrogel has been synthesis from acryloyl chloride modified olsalazine (as an azo crosslinker), which was next copolymerized with hydroxyethyl methacrylate and methacrylic acid. Two types of hydrogel DDSs which contained a single network hydrogel and interpenetrating network hydrogel as an enzyme-responsive and pH-responsive controlled release carrier for colon-specific 5-FU delivery was performed. The in vitro release of the 5-FU from these DDSs was carried out in PBS (pH 7.4 and pH 2.0) and rat colonic fluid (RCF). It was found that the 5-FU was released from hydrogels much faster in RCF. The results showed that these hydrogel DDSs could be potential drug carrier for colon-targeted delivery [75]. 

A very interesting nanocomposite hydrogel enzyme-prodrug systems (EPT) for local therapy have also been obtained [8]. EPTs were composed of a poly(DL-lactide-co-glycolide)-b-poly(ethylene glycol)-b-poly(DL-lactide-co-glycolide) (PLGA−PEG−PLGA) copolymer, LAPONITE, and β-galactosidase (β-gal). The nanocomposite gels can be easily injected locally, and the inherent enzyme activity of β-gal can be long-term preserved. It was found that a prodrug 5-FU-β-gal readily permeated into the interior space of gels and was converted into the active anticancer drug 5-FU. Importantly, a single local injection of SRH and a prodrug 5-FU-β-gal provided long-lasting antitumor activity in vivo without observable side effects [8].

Another interesting example is DDS for specific drug delivery within the colon. Enzymes present in the colon, such as dextranase, are able to break down DEX. These properties allowed the development of DDS in the form of a DEX hydrogel cross-linked with diisocyanate for the delivery of drugs in colon cancer. The biodegradation of such systems by Dextr has been investigated in vitro and in vivo. It was revealed that the drug was released without the presence of Dextr by diffusion, while in the presence of the enzyme, the drug was released as a result of hydrogel degradation. The mentioned hydrogel DDSs showed promising properties for drug delivery in the treatment of colorectal cancer directly at the target site [40].

## 5. Conclusions, Challenges and Prospects

In recent years, growing interest in biomedical hydrogels as drug-controlled release systems, has been observed. ERHs seem to be a very promising group of DDSs. However, despite the achievements that have been made in controllable cross-linking and de-cross-linking of hydrogels by utilizing enzyme-catalyzed reactions in the past few years, this strategy for hydrogel drug-controlled systems remain under development, yet.

The design and development of hydrogel-based drug-controlled systems meet several problems. Indeed, an ideal ERH as DDSs should be characterized by the below-mentioned features:

- possess a low viscosity of the hydrogel solutions to ensure a good injectability,
- allow a simple and efficient drug load,
- contain only biodegradable or bioresorbable and biocompatible components,
- possess good system stability,
- yield a low variability of drug release with a low initial burst,
- characterized high controlled drug release.

Despite the attractive physico-chemical and biological properties of ERHs, serious constraints do still exist. 

The main obstacles in the development of ERHs as DDSs are as follow:

- sometimes lack of complete drug release control,
- relatively frequent occurrence of the phenomenon of the drug burst release,
- sometime a high degree of complexity of the synthesis methods,
- relatively high cost of obtaining these biomaterials,
- sometimes the toxicity of the matrix forming materials and solvents used.

The decrease of variability and the improvement to provide a burst free, controlled drug release with predictable biological fate of a nontoxic carrier will be the main challenge for the future development of ERHs as DDSs. Many efforts are made in industry and academia to improve the current approaches. New hydrogels and approaches enter the preclinical phases, and one can be sure that ERH will gain further clinical importance within the next years.

## Figures and Tables

**Table 1:** Enzymes used in biomedical hydrogels synthesis.

Type of Polymeric Hydrogel	Enzymes	Refs
poly(allylamine) with acetyl protected dialanine	Elastase	[23]
hyaluronic acid/tyramine conjugate	HRP	[24]
DEX-tyramine linked by a urethane bond	HRP	[25]
DEX-tyramine linked by ester-containing diglycolic group	HRP	[25]
carboxymethylcellulose with phenol moieties by covalently incorporating tyramine	HRP	[26]
ALG with phenol moieties/tyramine	HRP	[24]
four-armed PEG terminated by 20-mer peptide	HRP	[27]
PGADA	HRP	[28]
marine derived oxidized ox-ALG, ADA, and Gel system/cross-linked Ca^2+^ and mTG	mTG	[29]
Gel	TGlu	[30]
multi-arm PEG	TGlu	[31]
cross-linked PEG	Tyr	[32]
Gel/chitosan conjugates	Tyr	[33,34]

**Table 2:** Enzyme-responsive biomedical hydrogels.

Type of Hydrogel	Effect of Action	Enzymes	Refs
Gel and DEX	Degradation of the polymer	α-Chym and Dextr	[38]
DEX cross-linked with diisocyanate	Degradation of the polymer	Dextr	[38,39,40]
Hydrogels consisting oligopeptide-terminated PEG and DEX	Degradation of the polymer	Dextr and Papain	[39]
poly(acrylamide)	Degradation by cleavage of cross-links	α-Chym	[41]
PEG	Degradation by cleavage of cross-links	Collagenase	[42]
pNIPAM grafted on DEX and a pNIPAM–N,N-dimethylacrylamide copolymer	Degradation by cleavage of cross-links	Dextr	[43]
PEG	Degradation by cleavage of cross-links	Elastase	[42]
PEG-oligopeptide-PEG telechelic block copolymers	Degradation by cleavage of cross-links	MMP-1	[44]
Multiarm-PEG	Degradation by cleavage of cross-links	MMP-1	[31]
multiarm vinyl sulfone-terminated PEG macromers and alpha-omega cysteine oligopeptides	Degradation by cleavage of cross-links	MMP-1	[45]
Pluronic and octapeptide multiblock copolymer	Degradation by cleavage of cross-links	MMP-2	[18]
pNIPAM-co-PAAc	Degradation by cleavage of cross-links	MMP-13	[46]
4-arm azido-terminated PEG and [alkyne]-GFLGK-[alkyne] or ([alkyne]-GFLG)2K peptide	Degradation by cleavage of cross-links	Papain	[47]
PEG	Degradation of the polymer	Papain	[39,48]
pNIPAM grafted on DEX and a pNIPAM–N,N-dimethylacrylamide copolymer	Degradation of the polymer	Papain	[39,48]
PEG	Degradation by cleavage of cross-links	Plas	[44]
PEG	Degradation by cleavage of cross-links	Plas	[49]
PHEMA/PEO and Gly-Gly-Leu tripeptyde	Degradation by cleavage of cross-links	Subtilisin	[50]
PEG	Degradation by cleavage of cross-links	TRYP	[49]
natural amino acid/aspartic acid copolymers cross-linked by tetrapeptide	Degradation by cleavage of cross-links	TRYP	[51]
PEGA	Morphology control	Dextr	[52]
PEGA	Morphology control	Elastase	[53]
Gly-Arg-Gly-Asp-Ser functionalised hydrogels	Morphology control	Glutathione-S- transferase	[54]
PEGA	Morphology control	MMP-1/12	[53]
PEGA	Morphology control	Thermolysin	[52]
PEGA	Morphology control	Thermolysin	[55]
PEGA	Morphology control	TRYP	[52]

**Table 3:** Hydrogel drug delivery systems activated by enzymes.

Drug	Type of Hydrogel	Enzymes	The Main Conclusions	Refs
Ins	N,N-diethylaminoethyl methacrylate and 2-hydroxypropyl methacrylate cross-linked with a polyacrylamide membrane	GO	The low pH of the membrane caused ionization of the amino groups present in the DDS, which led to the swelling of the HSBF and the membrane permeability to insulin increased.	[62,63]
Ins	DEX/chitosan	GO	*In vitro* insulin release can be modulated in a pulsatile profile in response to Gluc concentrations. *In vivo* studies validated that these formulations provided improved Gluc control in type 1 diabetic mice subcutaneously administered with a degradable nano-network. A single injection of DDS facilitated stabilization of the blood Gluc levels in the normoglycemic state for up to 10 days.	[64]
Ins	4-arm-PEG acrylic macromonomer	GO	The kinetics of hydrogel degradation and insulin release could be finely manipulated by tuning the concentration of H_2_O_2_ or changing the GO content and Gluc concentration. Of importance, an extremely low GO content was sufficient to afford a moderate insulin release at the hyperglycemic level, which would be beneficial for potential in vivo application.	[65]
Ins	poly(diethylaminoethyl-g-ethylene glycol)	GO	HSBF showed pulsatile reversible volume change when Gluc concentration varied between 0 and 2 g/L	[66]
Ins	poly(sulfadimethoxine)	GO	In Gluc concentration range of 0–300 mg/dl the ERH showed reversible sugar dependent swelling without hysteresis.	[67]
DOX	PEG-coated magnetic iron oxide nanoparticles	MMP	ERH were taken into cancer cells 11 times more efficiently than uncoated ones. These targeted nanocarriers were efficiently delivered and released DOX into the nuclei of HeLa cells within 2 h.	[69]
DOX	acrylate-PEG-PQ–PEG-acrylate conjugates, PEG-diacrylate and acrylate-PEG-RGDS	MMP	DOX loading efficacy was more than 97%. Drug in vitro release from obtained DDS was 60% and 36% after 4 days.	[70]
DOX	peptide-crosslinked nanogels (pNGs) -based on a dendritic polyglycerol	MMP	Stable conjugation of DOX at physiological pH and controlled drug release from pNGs were observed.	[71]
DOX	injectable polyamino acid-based nanogels (NGs)	Cathepsin B	DDSs were characterized with ~100 nm in size and 25 wt% drug loading. They content that became rapidly internalized in TNBC cell lines and displayed IC50 values comparable to the free form of DOX. NGs significantly inhibited lung metastases (in mouse model).	[72]
DOX	poly(ethylene glycol) hydrogel crosslinked via thiol-maleimide reactions	MMP	The hydrogel responded to both thermal and enzymatic stimuli in a local environment. DOX was loaded in the DDS with a high encapsulation efficiency.	[73]
DOX	disulfide cross-linked copolymer of 2-(dimethyl amino) ethyl methacrylate and PEG	GP	A relatively higher release of DOX was observed from the nanogels at pH 5.0 than at pH 7.4. DOX release was further accelerated in tumor simulated environment of pH 5.0 and GP.	[74]
5-FU	product polymerization of olsalazine-AC/ HEMA/ MAA	Enzymes contain in the rat colonic fluid	5-FU is locally released in colon part and the local high concentration of 5-FU induces necroptosis of colon cancer cells and circumvents cancer drug resistance.	[75]
5-FU	PLGA−PEG−PLGA	β-gal	A single local injection of SRH and a prodrug 5-FU-β-gal provided long-lasting antitumor activity in vivo without observable side effects.	[8]
Temozolomide	Triglycerol monostearate	MMP	Hydrogels effectively reduced the recurrence of temozolomide -resistant glioma after surgery and significantly enhanced the efficiency of temozolomide to inhibit glioma growth.	[76]

## Data Availability

Not applicable.

## Abbreviations

ADA—alginate dialdehyde
ADA-Gel—oxidized ALG, ADA and Gel hydrogel systems
ALG—alginate
Azored—azoreductases
CAT—catechol
α-Chym—α-chymotrypsin
DDS, DDSs—drug delivery system(s)
DEX—dextran
Dextr—dextranase
DOX—doxorubicin
EPT—hydrogel enzyme-prodrug systems
ERH—enzyme-responsive hydrogels
5-FU—5-flurouracil
β-gal—β-galactosidase
Gel—gelatin
Gluc—glucose
Gluc acid—gluconic acid
GO—glucose oxidase
GP—glutathione peroxidase
HPR—horseradish peroxidase
HSBF—hydrogels sensitive to bioactive substances
Ins—insulin
IPN—interpenetrating polymer network
MMP-1—metalloprotease 1
MMP2—metalloprotease 2
MMP13—metalloprotease 13
MMPs—metalloproteinases
mTG—microbial trans-glutaminase
NGs – nanogels
ox-ALG—oxidized alginate
PEG—poly(ethylene glycol)
γ-PGA—poly(γ-glutamic acid)
PGADA—poly(γ-glutamic acid)-dopamine
PHEMA—2-Hydroxyethyl methacrylate
PEGA—poly(ethylene glycol acrylamide)
Plas—plasmin
pNGs—peptide-crosslinked nanogels
pNIPAM—poly(N-isopro-pylacrylamide)
pNIPAM-co-PAAc—hydrogels composed of N-isopropylacrylamide (NIPAAm) and acrylic acid (AAc) with peptide cross-linkers
PLGA−PEG−PLGA—poly(DL-lactide-co-glycolide)-b-poly(ethylene glycol)-b-poly(DL-lactide-co-glycolide) copolymer
RCF—rat colonic fluid
SRH—stimuli-responsive hydrogels
TGlu—trans-glutaminase
T_trans_—sol-gel transition temperature
TRYP—trypsin
Tyr—tyrosinase
Ty-GG—tyramine-modified gellan gum
